# Atrial mapping during pulmonary vein pacing: a novel maneuver to detect and close residual conduction gaps in an ablation line

**DOI:** 10.1007/s10840-016-0159-9

**Published:** 2016-07-01

**Authors:** Jefferson Salas, Eduardo Castellanos, Rafael Peinado, Sergio Madero, Teresa Barrio-López, Mercedes Ortiz, Jesús Almendral

**Affiliations:** Electrophysiology Laboratory and Arrhythmia Unit, Hospital Monteprincipe, Grupo HM Hospitales, University CEU-San Pablo, Madrid, Spain

**Keywords:** Atrial fibrillation, Pace and map, Ablation, Pulmonary veins

## Abstract

**Background:**

Location of residual conduction gaps on ablation lines around pulmonary veins (PV) is challenging, and several maneuvers have been described. Atrial mapping during PV pacing—the “pace and map” maneuver—could localize gaps.

**Methods and results:**

We prospectively studied 209 patients undergoing PV isolation at a single institution over a 25-month period. In 24 (11.4 %) patients, 26 PV remained connected after an ablation line and an initial conventional gap closure protocol. The atrial side of the ablation line was mapped with the ablation catheter during PV pacing, and the earliest site was considered a gap site. Ablation at these gap sites resulted in bidirectional PV conduction block in 22 PV (85 %) in 21 patients (88 %), after 2.2 ± 1.6 radiofrequency applications and 8.2 ± 4.8 min. Early PV reconnection (≥20 min) occurred in 0 PV (0 %). During a mean follow-up of 12 ± 6 months, eight patients (33 %) had arrhythmia recurrences.

**Conclusions:**

The “pace and map” maneuver is a relatively simple and efficacious means to identify gaps in ablation lines around PV, focusing on the atrial rather than the PV side of the line. It could be considered among the ways to eliminate residual conduction gaps.

Circumferential pulmonary vein (PV) antral ablation with electrical isolation is the accepted cornerstone of interventional catheter-based therapy of atrial fibrillation (AF) [1]. Point by point radiofrequency (RF) ablation remains the most frequently used means to perform the procedure [2, 3]. This is accomplished by the deployment of successive RF lesions along an imaginary curvilinear line along the antrum of each individual PV or, encircling the PV on each side by a single ablation line, usually assisted by nonfluoroscopic navigation [2, 3].

If, after the completion of the lesion set around a single PV or the PV of one side, the PVs are not electrically isolated, this is usually due to one or more discrete gaps or small areas in the curvilinear line where electrical conduction remains. The identification of those gaps can be challenging, and several maneuvers, based on conduction into the PV [4–7], local excitability [8, 9], or image integration [10], have been described for that purpose.

We hypothesized that residual conduction gaps could be identified and subsequently ablated by mapping the atrial side of the ablation line during PV pacing and conduction out of the PV. The purpose of this study is to test the feasibility and efficacy of such a “pace and map” strategy.

## Methods

### Study design

Consecutive patients undergoing circumferential PV ablation with point by point RF ablation at our institution were prospectively considered potential candidates. After an initial ablation line along individual PV or along both ipsilateral PV, electrical conduction in both directions across the ablation line was studied. If one or more conduction gaps were present, they were searched for according to previously described maneuvers [4–6]. If after this initial standard gap closure protocol PV-left atrium (LA) or bidirectional conduction persisted, the “pace and map” protocol (see below) was performed.

### Patients

Patients undergoing circumferencial PV antral ablation to treat AF at our institution between April 2013 and May 2015 were included if they met the following criteria: (1) ablation was accomplished by point by point RF applications; (2) a deflectable sheath was used for the ablation catheter; and (3) the index procedure was the first PV ablation procedure in that patient. These criteria were met by 209 patients out of the 215 patients that underwent RF PV antral ablation first procedure during the study period. The pace and map protocol was applied in 24 patients, that constitute the study group. The study was approved by the Ethics Committee of the Institution. The clinical characteristics of the 24 patients of the study group are presented in Table 1.

**Table 1:** Clinical and imagine characteristics of the patients

	Pace and map (*n* = 24)
Age, year, mean ± SD	60 ± 18
Men, *n* (%)	22 (92)
Body mass index, mean ± SD	27.3 ± 2.9
Hypertension, *n* (%)	7 (29)
Diabetes, *n* (%)	2 (8)
Structural heart disease, any	4 (17)
Coronary, *n* (%)	1 (4.2)
Valvular, *n* (%)	0 (0)
Hypertensive, *n* (%)	1 (4.2)
Hypertrophic cardiomyopathy, *n* (%)	1 (4.2)
Amiloidosis, *n* (%)	1 (4.2)
Tachycardiomyopathy, *n* (%)	0 (0)
AF type	
Paroxysmal, *n* (%)	10 (42)
Persistent, *n* (%)	14 (58)
LA area, cm^2^, mean ± SD	29.4 ± 8.9
Normal LVEF, *n* (%)	24 (100)

*AF* atrial fibrillation, *LA* left atrium, *LVEF* left ventricular ejection fraction

### Mapping and ablation procedure

Transesophageal echocardiography was performed either at the beginning of the procedure or on the day before to exclude a left atrial (LA) thrombus. Each patient gave written informed consent prior to the procedure. The procedures were performed under general anesthesia, and without stopping coumadin if the patient was on this agent. Mapping and ablation was performed, if possible, during sinus rhythm; performing electrical cardioversion after catheters was advanced to the LA if the patient was in AF. If AF recurred, cardioversion was performed again after the ablation lines were completed.

Two catheters were advanced to the LA through a single transeptal puncture, a 20-pole circular mapping catheter (Reflexion Spiral, St Jude Medical Inc., St. Paul, Minnesota) and a 3.5 or 4 mm irrigated ablation catheter (EZ Steer SF, Biosense Webster, Diamond Bar, California, or Tacticath Quartz, St Jude Medical Inc., St. Paul, Minnesota). A third, standard quadripolar catheter was used for reference. Heparin was infused to maintain a target ACT of ≥350 s. A LA and PV geometry was created, mostly with the circular catheter, using the ablation catheter for fine tunning of specific areas. A temperature probe (Sensitherm, St. Jude Medical, Inc., St. Paul, Minnesota) was introduced in the esophagus and carefully positioned at the level of the PV to monitor esophageal temperature during RF applications.

RF was delivered in the unipolar mode between the ablation catheter and a back-plate distant skin electrode. RF parameters were set at a temperature of 43 °C and a power of 25–30 W except for the posterior wall where the power was decreased to 20–25 W. The duration of each application was generally of 45 s, shorter if the local bipolar electrogram voltage decreases markedly in the first seconds of the application, and up to 60 s in cases of minor decrease of local electrogram voltage. When using a contact force sensing catheter, a contact force between 10 and 40 g was aimed, and applications were stopped if the “lesion size index” achieved 5 in the posterior wall or 5.5 elsewhere, before reaching 60 s. Applications near the esophagus were stopped if the temperature probe reached 38.5 °C, and further applications at such areas were adapted to the esophageal anatomy to prevent as much as possible a temperature raise.

The ablation catheter was navigated with a deflectable sheath in all cases, either a manual sheath (Agilis, St Jude Medical, Inc., St. Paul, Minnesota) or a robotic sheath (Artisan, Hansen Medical, Inc., Mountain View, CA, USA). A curvilinear line was drawn around the antrum of each PV or the PV on each side (at the discretion of the operator), over the previously created 3D geometry as a reference for RF lesions. The ablation was performed as follows: the catheter was placed on the drawn line, and RF applications were deployed “point by point”, with the parameters previously described, and marked with a 4 mm tag on the geometry. After each RF application, the catheter was advanced along the line to a contiguous site where an electrogram was observed, to deploy a subsequent contiguous application. The circular catheter was kept inside each PV during applications at each antrum moving it to the neighboring PV as applications along the line arrived to its antrum. After an ablation line around ipsilateral PVs was completed, bidirectional conduction between LA and the treated PVs was analyzed. If bidirectional conduction was absent, it was reevaluated after a waiting time of ≥20 min. Adenosine was not routinely used.

If electrical conduction remained, an initial standard gap closure protocol was undertaken during sinus rhythm or atrial pacing. Two basic principles were used to localize conduction gaps, as previously described [4–6]. (a) A precise localization of the earliest PV potential from the circular catheter at the PV antrum, indicating impulses enter the PV at that area. The circular catheter could be moved more antral in order to be more precise. (b) Dragging the ablation catheter along the ablation line to identify sites with a higher voltage and morphologic characteristics of the electrograms, such as fractionation that could be indicative of conduction at those areas. Further RF applications were deployed at sites were gaps had been suspected. The “intensity” of this standard gap closure protocol was limited in that applications with an increased power, inside the PV close to its tubular portion or in close proximity to the esophagus with elevation of its temperature were not performed at this time, and only performed if the pace and map protocol failed.

### “Pace and map” maneuver

**Fig. 1:**
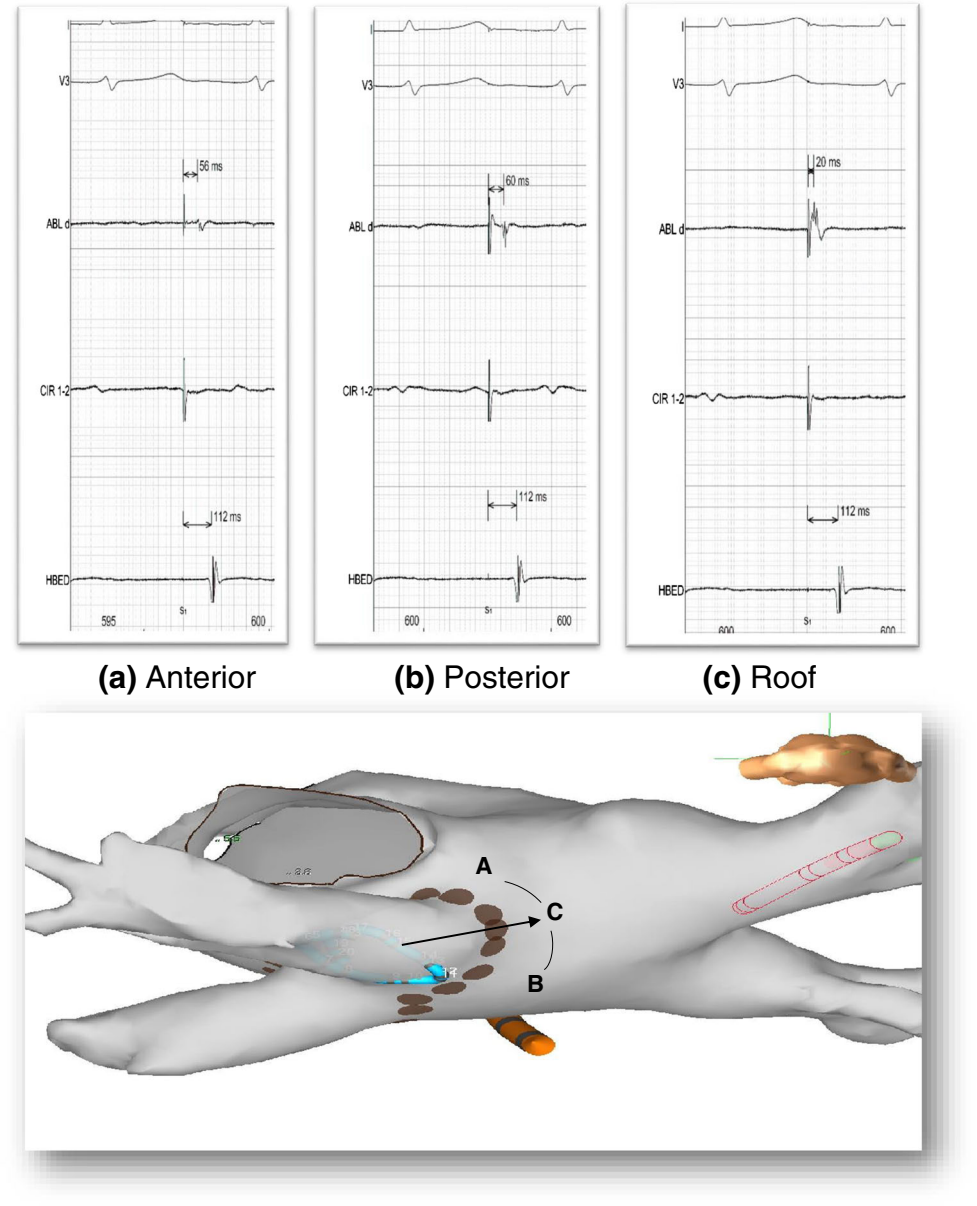
Superior panel. Representative example of tracings during a pace and map maneuver localizing a gap on the roof of the left PV. Each tracing shows one surface ECG, the distal bipolar recording from the ablation catheter, one recording from the circular (pacing) catheter, and a right atrial electrogram. Note that the activation time at the ablation catheter is shorter at the roof (site **c**) than at the anterior or posterior PV wall (sites **a** and **b**), indicating a gap at the roof. Inferior panel: schematic representation of location of sites **a**–**c** superimposed on the 3-D atrial geometry. The *arrow* represents conduction exiting from the PV through the gap. Ablation at site **c** resulted in PV isolation

**Fig. 2:**
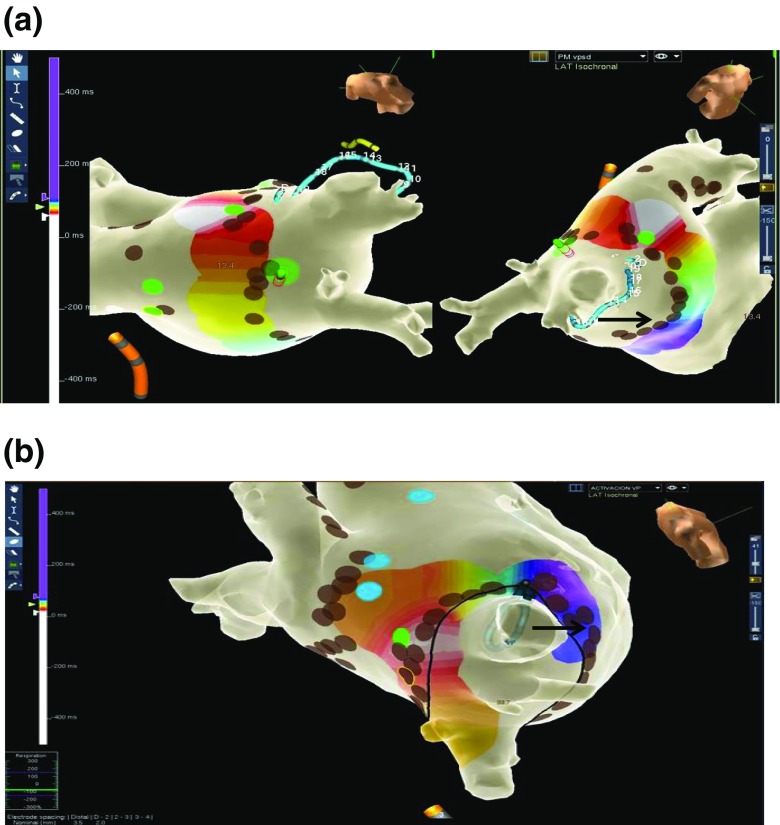
Representative examples of color-codded activation maps showing one gap on the ablation line around PV. All panels depict the LA anatomy with an activation map obtained with a limited number of points around ipsilateral PV, during PV pacing. The brown dots represent RF applications sites from the initial ablation line and the green dots depict the successful RF application based on the pace and map maneuver. The black arrows indicate earliest sites inside the PV during sinus rhythm. Panel **a**: Gap on the roof of the right superior PV. The activation map, was obtained during right inferior PV pacing. On this site, one radiofrequency application resulted in isolation of both right PV. Panel **b**: Gap on the posterior aspect of the right superior PV

**Fig. 3:**
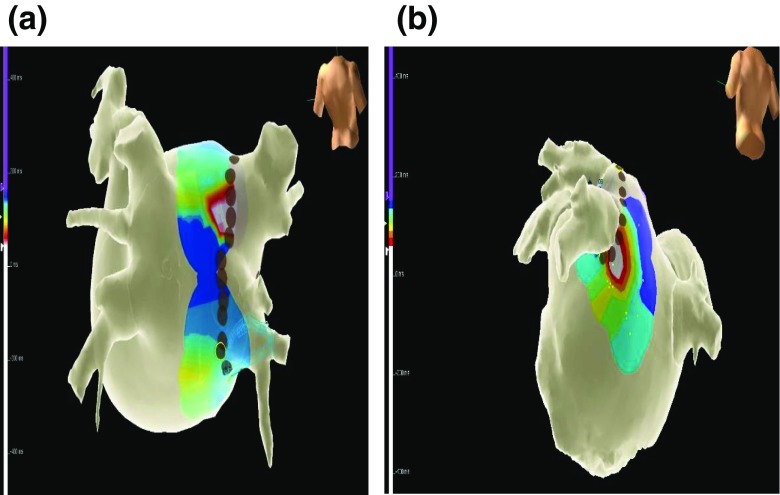
Representative example of a color-coded activation map showing two gaps during ablation of the both left PV. The map was obtained during left superior PV pacing. **a** The first gap was on the roof of the left superior PV. **b** Second gap was on the floor of the left inferior PV. RF application at this second gap resulted in left superior PV isolation

Electrophysiological mapping indicates that, if pacing is performed inside the PV and there is a single conduction gap between the PV and the LA, the earliest electrical breakthrough into the LA will be located at the site of the conduction gap. Thus, bipolar continuous pacing at a cycle length of 600 ms and an output of 10 mA (2 ms pulse width) were performed from the circular catheter at a site inside the PV (to avoid direct LA capture) but with consistent PV capture. Since any PV pacing site will produce the same result, the PV pacing site was selected taking into consideration: (1) the site is well inside the PV as shown in the 3-D navigation system; (2) during sinus rhythm, the activation time is as late as possible as compared with the other PV recordings; and (3) there is consistent pacing capture. The pacing site was chosen during PV pacing; the ablation catheter was dragged along the atrial side of the ablated line, looking for sites that are clearly in the LA and out of the ablated line but as close as possible to it and where an electrogram could be recorded. Conduction time between the pacing stimulus and the local electrogram is measured at each mapped site. The site with the earliest activation under these circumstances was identified (Fig. 1) and considered the conduction gap. There are two steps in the process: first, distant sites along the ablation line are mapped to detect the general area where the gap is located, and later fine tuning of close sites determines the site with earliest activation. In the last 14 cases, a color-coded representation of the local activation time of the mapped sites was used to assist in a faster localization of the earliest activation site (Figs. 2 and 3). In such cases, the window of interest starts immediately after the stimulus artifact, which is used as reference, and usually ends at the QRS onset, using the automatic color coding system. RF applications were delivered at the identified conduction gaps or immediately adjacent sites during PV pacing until there was PV-LA conduction block. If this did not occur, two possibilities were contemplated: (1) the area already ablated remains as the site with the earliest activation, that would indicate insufficient ablation; and (2) the area already ablated is now activated later, and a different area is now the site with the earliest activation, a second gap was suspected, and the atrial side of the ablated line was explored again looking for a different electrical breakthrough into the LA (second gap, Fig. 3). The maneuver was abandoned if the gap site and adjacent sites remained as the earliest area, and the investigator judged that appropriate ablation of that area has already been accomplished.

### Further analysis of gaps

For descriptive purposes, gap location on each PV was divided into the following segments: anterior, posterior, roof, carina, and floor.

We intended to obtain an idea as to if the identified gaps with the “pace and map” maneuver were located in close proximity to what could have been considered a likely gap with the standard gap closure protocol. For that purpose, the electroanatomical map was “scaled” (if needed) to an anatomical representation, and gap sites were considered concordant if they were located in the same PV segment or discordant if they were in separate segments. In order to quantify this relationship, a linear distance was measured between the gap site according to the “pace and map” maneuver and the gap site according to a standard localization during sinus rhythm.

Electrograms at gap sites were analyzed and classified as with a single component or fractionated.

### Statistical analysis

Quantitative variables are presented as mean ± SD, and qualitative variables are presented as absolute numbers and percentages.

## Results

### Procedural results

The initial ablation line combined with the initial standard gap closure protocol resulted in bidirectional electrical isolation of all PV in 185 of the 209 patients (88.6 %). The pace and map protocol was attempted in the remaining 24 patients (11.4 %), treating 26 PV (3.4 % of all treated PV) that remained electrically connected with the LA.

Tables 1 and 2 describe the clinical and procedural variables of the study group. No procedural complications occurred in the study group.

**Table 2:** Procedural and follow-up variables

	Pace and map (*n* = 24)
Procedural duration, min, mean ± SD	240 ± 36
Agilis sheath, *n* (%)	11 (46)
Hansen robotic sheath, *n* (%)	13 (54)
Contact force sensing catheter, *n* (%)	19 (79)
Sinus rhythm at procedure onset, *n* (%)	9 (37.5)
No. RF applications, mean ± SD	56 ± 10
Left PV: no. RF applications, mean ± SD	26 ± 7
Right PV: no. RF applications, mean ± SD	31 ± 8
Early PV reconnection (per PV)^a^, *n* (%)	0 (0)
Fluoroscopy time, min, mean ± SD	32 ± 11
Periprocedural complications, *n* (%)	0 (0)
Follow-up, months, mean ± SD	12.7 ± 6.4
Atrial fibrillation recurrence, *n* (%)	5 (20)
Atrial tachycardia/flutter recurrence, *n* (%)	3 (12)

*PV* pulmonary vein, *RF* radiofrequency

^a^Only in PV after pace and map was performed

### Pace and map protocol

The results of the pace and map protocol are summarized in Table 3. Overall, 26 PV were treated in 24 patients, a single gap was identified in 22 patient, and in 2 patients was identified two gaps, and ablation resulted in PV isolation in 22 PV (85 %) with a mean of 2.2 ± 1.6 RF applications, in 21 patients (88 %). Illustrative examples are shown in Figs. 1, 2, and 3. The mean time required for gap location and ablation was 8.2 ± 4.8 min per PV. In all instances when PV-LA conduction block developed, there was already LA-PV conduction block as shown when pacing was terminated.

**Table 3:** Pace and map results

	No. cases	No. success (%)
Overall, patients	24	21 (88)
Overall, PV	26	22^c^ (85)
No. RF applications to close gap, mean ± SD	^b^	2.2 ± 1.6
Duration of gap mapping and ablation, min, mean ± SD	8.6 ± 5.9	8.2 ± 4.8
PV		
Left superior	2	1 (50)
Left inferior	3	3 (100)
Left common trunk	1	1 (100)
Right superior	15	12 (80)
Right inferior	5	4 (100)
Gap location	^b^	24
Roof		9 (37.6)
Carina		4 (16.6)
Posterior		6 (25)
Anterior		4 (16.6)
Floor		1 (4.2)
Concordance between gap location and earliest PV EG in the circular catheter during pace	^b^	
Concordant^a^		6 (25)
Discordant		18 (75)
Distance: gap to site in front of earliest PV EG (mm) voltage, ms, mean ± SD		20.4 ± 9.6
		0.22 ± 0.12
EG width, ms, mean ± SD		34.1 ± 15.3
EG morphology at the gap	^b^	
Single component		18 (75)
Fractionated		6 (25)

*EG* electrogram, *PV* pulmonary vein, *RF* radiofrequency, *SR* sinus rhythm

^a^Concordant: when gap location as determined by the circular catheter during sinus rhythm and by the pace and map maneuver were in the same PV segment. Otherwise, gap location was considered discordant

^b^Only applicable in cases with gap closed successfully

^c^In one vein, the gap, was successfully identified, and RF application resulted in PV isolation, but it was stopped because of increase in esophageal temperature, and PV conduction returned

Gaps were present more often (50 % of the gaps) in the right superior PV (Fig. 2) with similar prevalence in the remaining PV and had multiple locations within the anatomy of the antrum. Gaps were concordant with a likely gap during sinus rhythm (SR) in 6 PV (25 %) and discordant in the remaining 18 (75 %). The mean distance between the gap and a likely gap during SR was 20 ± 9 mm (range 4–39 mm). The morphology of the electrogram at gaps sites had a single component in 75 % of cases and was fractionated in 25 %.

In the reevaluation ≥20 min after PV isolation, reconnection had occurred in zero patient (0 %) in the study group, Table 2.

In the four PV that remained connected after the pace and map maneuver, further attempts were made to localize and ablate the gaps with the standard method. Two PV could be isolated by RF applications sligthly inside the ablation line; in another PV, unidirectional LA-PV block was obtained and the remaining PV persisted connected.

### Clinical follow-up

Patients were followed up for a mean of 12 ± 6 months. Five patients (20 %) have recurred with AF, and three (12 %) have recurred with atrial tachycardia/flutter (Table 2). Two of the three patients in whom the pace and map maneuver was unsuccessful had arrhythmia recurrences.

## Discussion

The main findings of this prospective, nonrandomized, noncomparative, “proof of concept” study are as follows: (1) conduction gaps that remain after point by point circumferential antral PV radiofrequency ablation and an initial standard gap closure protocol can be identified by the application of electrophysiologic mapping principles at the outside of the ablation line during PV pacing (“pace and map” maneuver); (2) ablation at those sites results in bidirectional LA-PV conduction block in the majority of cases; and (3) gaps located with this maneuver are frequently discordant with and at a considerable distance from the likely gap during LA-PV conduction as recognized by the site in the line in front of the earliest PV site.

### Difficulties in conduction gap identification during PV ablation

PV is known to have muscle sleeves that run from the LA into the distal PV [11]. Electrophysiologic principles can then be used to localize such sleeves by mapping electrical activation of so called PV potentials during LA to PV conduction in sinus rhythm or atrial pacing. In fact, such principles allowed the identification and subsequent ablation of multiple and discrete breakthroughs into the PV [12], and this same principle is also used to localize conduction gaps after a circumferential ablation line is attempted [4].

Since the shift in ablation strategies to target the atrial tissue located in the antrum rather than the PV itself [1], localizing conduction gaps has become more complicated for a number of reasons: (1) the course of muscle sleeves may be circular or spirally oriented with additional bundles longitudinal or obliquely oriented [11]; and (2) PVs are funnel-shape structures, and PV ostia are oblong in shape with an anteroposterior dimension less than the superoinferior dimension [13], so a circular mapping catheter does not always fit inside the PV with an orthogonal angle or at the right level. Thus, gap detection is not always as simple as identification of the site on the ablation line in front of the earliest PV potential.

### Methods proposed for identification of conduction gaps in the ablation line

In this context, several maneuvers have been proposed for conduction gap localization and ablation. Liu et al. compared an approach that targeted the site with the earliest electrical activation on the circular catheter inside a PV with an approach that delivered further applications at the previous ablation line, at sites found to be activated early just inside the ablation line [4]. Acutely, the former was successful in all 50 patients and the latter in 48 of 50 patients, but the latter approach resulted in a lower arrhythmia recurrence rate over a mean follow-up period of 12 months. A subsequent study of the same group with similar design included the exclusive use of the ablation catheter to map just inside the ablation line, and both groups were found to be equally successful acutely and during follow-up [6]. A characteristic morphology of the local electrogram of the ablation catheter with multiple components but without an isoelectric line was found to predict 85 % of conduction gaps on the ablation line at which further ablation resulted in PV disconnection, although it was less useful during AF as compared to sinus rhythm [5]. More recently, an electroanatomical map inside the PV was advocated for gap localization and used in 18 patients; it was successful at closing gaps in 24 out of 26 PV [7].

A different approach was reported by two groups on the same year; they explored if gaps could be identified by a preserved excitability during pacing at the previously ablated line [8, 9]. One group performed bipolar pacing at 10 V/2 ms with the ablation catheter that was moved along the ablated line, and a gap was considered to be present at sites with capture. Gap identification and closure, as performed with this method, were considered successful in 95 % of 147 patients and confirmed with a circular catheter in 94 % [8]. In a group of 30 patients, this same approach successfully produced electrical isolation of 57 of 60 PV pairs [9]. A subsequent randomized study of this group showed that loss of excitability along the ablated line resulted in PV isolation in 98 % of 50 left PV pairs and 96 % of 50 right PV pairs, and these patients had lower arrhythmia recurrence rate than the control group [14].

An even different approach have been described that with the use of cardiac magnetic resonance with delayed enhancement was able to identify conduction gaps prior to redo procedures that matched electrical gaps in 79 % of PV [10].

### The “pace and map” maneuver

Pacing within the PV consistently results in capture and impulse propagation out of the LA [15], and some groups use exit block (in addition to entrance block) as a requirement for demonstration of PV isolation [1]. Localization and ablation of conduction gaps on the atrial side of an ablation line during PV pacing have certain theoretical advantages: (1) conduction is expected to be less circuitous and/or complex than on the PV antrum itself, and more amenable to electrophysiological mapping principles; (2) electrogram amplitude and morphology is expected to be less abnormal than in the ablation line itself; and (3) strict catheter stability is not particularly required during the mapping process.

The pace and map maneuver as such is simple and lasted a mean of 9 min including RF application time (Table 3). Although electroanatomical mapping is not strictly necessary and was not used in our initial cases, we found color-coded representation of electrical activation particularly useful and sped up the process. The maneuver was efficacious in the majority of cases and located the gaps with precision since only a mean of two RF applications were needed. Of note, once exit block was observed, entrance block was present in all cases. However, it has to be realized that the method was applied after failure of a conventional gap closure protocol. As such, the value of the pace and map method as a primary gap closure maneuver was not tested since it was felt that it should initially be employed in cases resistant to conventional maneuvers. The gap site was at a considerable distance (mean 20 mm, Table 3) from the likely gap as detected during sinus rhythm, probably expression of the tortuous anatomy of PV sleeves and attesting to the uniqueness of this maneuver, that locates “a different gap site for the same gap”. This situation may be analogous to what is seen with slant accessory pathways having an atrial insertion not in front of their ventricular insertion. Probably expression of this same phenomenon is that the electrogram at successful sites was rarely fractionated.

A potential advantage of “pace and map” over maneuvers based on gap localization inside the ablation line is that RF applications will be delivered at or outside the original ablation line, thus decreasing the possibility of PV stenosis; it has been recognized that, with the conventional approach, RF applications at the tubular part of the PV are occasionally needed [7]. Also, in cases with unidirectional entrance block into the PV, these maneuvers cannot be applied [15, 16]. A potential advantage over maneuvers based on excitability is that they require catheter stability at all sites of the ablation line to check for pace capture, and increase significantly procedure time (a mean of >40 min in one study) [14].

The maneuver was unsuccessful in four PV in three patients. The reasons for that are unknown. An increase in atrial muscle thickness, edema from prior applications, or inadequate mapping precision can potentially explain such failures.

The observation of a low acute reconnection rate and a recurrence rate, that can be considered on the lower side of what is usually reported, also suggest clinical usefulness of this maneuver.

### Limitations

Our PV isolation rate (89 % of patients and 96 % of PV) with initial conventional maneuvers is rather low. Some studies report 100 % PV isolation rate, although not all check for bidirectional block. However, this is not universal, and some other well performed and controlled studies report similar results to ours, for example, Piorkowski et al. reported 88 and 78 % PV isolation rate with and without steerable sheath [17], and Eitel et al. reported 95 and 94 % PV isolation rate [8]. We also recognize that our initial gap closure protocol had a limited “intensity” having the expectation of performing the pace and map maneuver. Without such expectation, we had probably insisted more in the conventional maneuvers and had a better PV isolation rate.

The pace and map maneuver cannot be performed during AF. However, this was not a problem in our study, since 13 of the 14 patients of the study group in whom the procedure was initiated in AF were successfully cardioverted at the initiation of the procedure, and the remaining patient could be cardioverted after the initial ablation of the PV. In addition, it cannot be performed if there is unidirectional exit block, but we did not find such a situation in our study population.

The precise width of a conduction gap can be difficult to determine. The earliest site is considered the gap site and neighboring sites with later electrical activation time are considered part of the line of block. However, the gap may be wider and conduction may be occurring (although with slightly lower velocity) at sites in the vicinity of the earliest site. Such situation may be difficult to recognize and could actually be the reason for some of our failures. It is conceivable that such situation would result in closer isochrones in the activation map, and be identified, but we need more experience to clarify this subject.

A potential pitfall should be stressed; the ablation catheter could record short activation times if it is placed on the antral side of the PV, recording in fact antral, or PV potentials rather than atrial potentials during PV pacing. This could be totally misleading in gap localization, but it can be easily detected by moving the catheter out of the ablation line to its atrial side; in fact, a big change in activation time when such maneuver is performed speaks against a conduction gap at that site.

The study was not designed to compare the pace and map maneuver with other maneuvers to locate conduction gaps and was performed after initial conventional maneuvers have failed. As such, and despite the solid electrophysiologic basis of the maneuver, this study could be considered a proof of concept study, and we felt that this situation required a design in which conventional maneuvers had been used first. Further studies are necessary to compare different maneuvers and to test the utility of the pace and map as the first maneuver in gap location.

The ablation catheter (although with irrigated tip in all instances) was not the same in all procedures, and some were contact force sensing catheters, reflecting the changing trends in our laboratory during the study period. Similarly, although deflectable sheaths were used in all cases, some were manual and other robotic. Although these differences could have affected the incidence of gaps at the ablation line, it is unlikely that they could have influenced the performance and success of a method to detect and close conduction gaps once they are present.

All except one of our PV with gaps had a single gap. The method could theoretically detect more than one gap before or after closure of the first gap (Fig. 3) depending upon differences in conduction time, but this was only tested once in our study population.

## Conclusions

We describe a maneuver to detect conduction gaps on the atrial side of an ablation line around PV based on PV pacing and conventional mapping (“pace and map” maneuver). It was found useful in the majority of a consecutive series of 24 patients in whom conventional maneuvers had failed. Further studies are necessary to compare this maneuver with the other previously described to complete PV electrical isolation.
